# Prevalence and correlates of depression and psychological distress among garment factory employees in Hambantota district, Sri Lanka

**DOI:** 10.1186/s40359-025-03137-6

**Published:** 2025-07-17

**Authors:** Lasith Obadaarachchi, Amila Isuru, Suwin Hewage, Manuja Vipuladasa, Shehan S.Williams

**Affiliations:** 1 District General Hospital Chilaw, Puttalam, Sri Lanka; 2 Faculty of Medicine and Allied Sciences, Rajarata University of Sri Lanka, Anuradhapura, Sri Lanka; 3 Independent researcher, 603/10A, Siri Parakum Mawatha, Mulleriyawa, Colombo, Sri Lanka; 4 Regional Directorate of Health Services Hambantota, Hambantota, Sri Lanka; 5 Faculty of Medicine, University of Kelaniya, Kelaniya, Gampaha, Sri Lanka

**Keywords:** Garment industry, Depression, Correlates, Psychological morbidity

## Abstract

**Background:**

Garment factory employees have been identified as a high-risk population for developing depression in Asian countries. Depression is recognised as a potentially reversible risk factor for low productivity in the garment factory workforce. Therefore, identification and treatment of depression in garment factory workers can improve productivity and their quality of life in general. The study aimed to determine the prevalence and correlates of depression in garment factory workers in the Hambantota district, Sri Lanka.

**Methods:**

This is a cross-sectional descriptive study. The calculated sample size was 381, and a multistage random sampling method was used. Culturally validated General Health Questionnaire-12 and Beck Depression Inventory–II were used to screen for psychological morbidity and depression, respectively.

**Results:**

The majority of 381 employees were females (*n* = 325, 83.3%). The mean age was 32.9 years (SD—10.45 years). The prevalence of depression in the study sample was 16.80% (95% CI: 13.04–20.55%). Depression was associated with the presence of a chronic medical condition (OR-3.51, 95% CI:1.61–7.67), family history of psychiatric illness (OR-3.03 95% CI-1.11 to 8.26), history of deliberate self-harm (OR-10.79 95% CI-4.68 to 24.89), history of psychiatric illness (OR-6.12, 95% CI-2.39-15.73), and being divorced or separated from their partner. The only job-related factor that correlates with depression was working extra duty hours (OR-1.74, CI 1.01–3.02).

**Conclusions:**

The prevalence of depression among garment factory employees in Hambantota district is higher than the national average. However, it is relatively lower compared to garment factory populations in other developing Asian countries.

## Introduction

The garment manufacturing industry employs thousands of women in low and middle-income countries. In Sri Lanka, over 300,000 persons are employed in this industry, and the vast majority of them are women [1, 2]. The contribution by the females employed in this sector has enabled the sector to be Sri Lanka’s largest export earner since 1986 [3]. According to the Central Bank of Sri Lanka in 2022, the textile and garment sector accounted for 45.4% of the total export earnings of Sri Lanka [4]. Developing Asian Countries have been the main ready-made garment product exporters to the world. The lack of alternative job opportunities and the fact that it does not require prior skills make it a favourable job option to many, particularly women [5].

Considering the role played by these female employees, who at times work under tough social, financial and physically and emotionally demanding circumstances, understanding the prevalence and risk factors of depression among them is important. Most of the garment factories in Sri Lanka are clustered in urban areas of each district. As a result, there is labour migration from rural areas to urban areas leading to varied social adversities. Their pay is comparatively low, they usually have to work long hours and live in less than satisfactory accommodation [3]. Poor working conditions are associated with depression among textile factory workers [6].

Depression affects employees’ quality of life and substantially reduces working capacity at an individual level. From the point of view of employers, it leads to absenteeism and loss of productivity [7]. The economic impact may be higher in developing countries like Sri Lanka where the economy depends largely on labour-intensive manufacturing industries like the garment industry. Previous studies in other Asian countries have demonstrated a high prevalence of depression in this population, with rates ranging from 18 to 26% [5, 8–10].

Research on depressive disorder among garment factory employees in Sri Lanka is not documented. A few studies that have been done look at burnout and general psychological distress as measured by the General Health Questionnaire (GHQ) [11, 12]. Another study reported suicidal ideation in 9.8% and substance use of 3.7% in female garment factory workers between the ages of 16–49 [13].

The objective of this study was to assess the prevalence and correlates of depression in garment factory employees in the Hambantota District, Sri Lanka. Hambantota district has a population of around 600,000 people and is located on the southeastern coast of Sri Lanka. Considered predominantly to be comprising rural agricultural communities, the district has seen rapid urbanization in the last two decades.

## Methods

### Study design and sampling

This was a cross-sectional descriptive study. The study sample was selected from employees of garment factories in the Hambantota district, Sri Lanka.

The calculated sample size was 381 based on the findings of a research on the prevalence of depression among garment factory workers of 20.9% [10], accounting for a margin of error of 5%, alpha error of 5% and a 10% dropout rate.

Multi-stage sampling method was used in recruiting the participants for the study. Three subdivisions, Tissamaharama, Tangalle and Hambantota, where there were more garment factories, were identified in the district. Out of 13 garment factories with over 100 employees in the sub-districts, two garment factories were randomly selected from each sub-division. A proportionate number of participants from each garment factory were randomly selected from the employee register.

Garment factory workers who were 18 years or older were included in the study and those who had been employed for less than 1 month were excluded from the study as it was assumed that the new recruits within their first month of employment may experience adjustment symptoms which can be wrongly identified as depression due to overlap of symptoms of the two conditions.

### Data collection

Data collection was performed by two psychiatrists and a medical officer in occupational health who has training in mental health. A specifically designed data collection form was used to record general information and information related to potential correlates of depression.

The General Health Questionnaire-12 (GHQ-12) validated for the Sri Lankan population was used to screen for psychological morbidity. It is a reliable and valid instrument with a stable factor structure across different samples and cultures and its brevity gives it an added advantage [14]. The standard scoring (0-0-1-1) was used and the cut -off level of > 1 was used as it had been suggested to be optimal when using the standard scoring method to detect the presence of psychological morbidity [15, 16].

The Beck Depression Inventory-II (BDI II) was used to screen for depression and it was administered to all individuals who were screened positive for GHQ-12. BDI II is a widely used self-rating screening tool for depression with good psychometric properties, good test-retest reliability, high internal consistency, good construct and concurrent validity and discriminant validity [17, 18]. It has 21 items, each scored on a 4-point scale according to the degree of severity. It has been validated for the Sinhala speaking Sri Lankan population, and a score of > 15 was taken as indicative of depression [19].

### Data-analysis

Demographic and employment-related characteristics were analysed as frequencies and percentages. Comparison of dichotomous variables was performed by Pearson’s chi-squaretest. Depression status was presented as a dichotomous variable. Multiple logistic regression analysis was performed to identify the independent predictors of depression and psychological morbidity of garment factory workers. Bivariate analysis was conducted to identify potential predictor variables for the final fitted model. The adequacy of the final fitted model was assessed with Hosmer and Lemeshow goodness of fit test. A type 1 error of 0.05 was considered in all significance analyses. Data analysis was performed by the IBM, SPSS version 22.0.

#### Ethical approval

was granted by the ethics review committee, faculty of medicine, University of Ruhuna. Administrative permission was obtained by the Regional Director of Health Services, Hambantota, and from the respective managers of garment factories.

#### Ethical approval

was granted by the Ethics Review Committee, Faculty of Medicine, University of Kelaniya, Sri Lanka (P/05/01/2020). Informed written consent was obtained from all interviewees. Employees who screened positive for GHQ or BDI II were referred to counsellors, and if required, referred to the nearest mental health team.

## Results

### Baseline characteristics

A total of 381 garment factory workers were included in the analysis. The sample was predominantly female (84.0%). The age of participants ranged from 18 to 68 years with a mean of 32.9 (SD = 10.5) years. All the workers were from the Sinhala ethnicity and almost all (99.2%) identified themselves as Buddhists, with the remainder being Catholic. More than half of the workers were married at the time, while 34.4% were neither currently nor previously married (See Table 1).

**Table 1:** Frequencies of marital status categories among the sampled garment factory workers

Marital status category	Number (percentage out of total)
**Currently married**	203 (53.3%)
**Never married**	131 (34.4%)
Currently not in a relationship	67 (17.6%)
Currently in a relationship	64 (16.8%)
**Previously married**	47 (12.3%)
Separated	24 (6.3%)
Widowed	14 (3.7%)
Divorced	9 (2.4%)
**Total**	**381 (100.0%)**

Sewing Machine Operators (57.7%) followed by Helpers (20.7%) and Quality Controllers (10.2%) constituted the main job categories covered among the factory workers. Over 95% of the workers were monthly or weekly wage earners. A small percentage of the Machine Operators and Helpers were either paid daily or according to the amount of work done (See Table 1). Almost three-quarters (74.3%) of the respondents were working at a factory in their own hometown. Over 86% of respondents own a permanent job contract.

In response to health-related questions in the study, 10.0% of the sample identified themselves as having chronic physical illnesses, while 5.0% have had a history of depression. Almost eight per cent of the sample (7.9%) revealed that they have previously attempted suicide. Family history of mental illness was noted by 5.8% of the responding garment factory workers.

### Prevalence of psychological morbidity

As the screening tool for psychological morbidity, GHQ-12 scores of the garment factory workers showed the full range of values from 0 to 12, with a mean of 1.75 (SD = 2.55). Of the 381 participants, 132 had a score of over 1. Therefore, based on the categorisation of GHQ-12 scores above 1 as being positive for psychological morbidity, the estimated prevalence of psychological morbidity in the study was 34.65% (95% CI: 21.04–48.25%).

### Predictors of psychological morbidity

Before fitting a logistic regression model for psychological morbidity (as screened by the GHQ-12), potential predictors were assessed using bivariate analysis. The predictors considered in this regard were: age, gender, highest education level, marital status, number of dependents, accommodation type, travel time from residence to factory, whether factory is in their hometown or not, job title, income category, salary interval, level of work experience, whether or not engaging in extra duty work, employed full-time or part-time, being a permanent or casual worker, amount of time spent daily on watching television, amount of time spent daily on social media, presence of substance use, currently being pregnant or not, presence of a chronic physical illness, history of depression, history of attempted suicide and family history of mental illness. Out of which, only six variables i.e., marital status, salary interval, presence of a chronic physical illness, history of depression, history of attempted suicide and family history of mental illness showed individual statistically significant relationships with psychological morbidity (See Table 2). When a multiple logistic regression analysis was attempted using these six predictor variables, two (marital status and family history of mental illness) became non-significant. Therefore, only four of the factors assessed in the study were found to be independent predictors of psychological morbidity (See Table 3).

**Table 2:** Results of bivariate analysis for associated factors for psychological morbidity

Potential Predictor Variables Considered	Unadjusted Odds Ratio (95% Confidence Interval)	Statistical Significance
**Age**	0.99 (0.97 to 1.01)	*p* = 0.397
**Gender** Female compared to male	1.21 (0.67 to 2.17)	*p* = 0.531
**Highest level of education** Above Ordinary Level compared to up to Ordinary Level	1.33 (0.84 to 2.10)	*p* = 0.220
**Marital status** Never married compared to currently married Separated, divorced or widowed compared to currently married	1.54 (0.97 to 2.46) 2.61 (1.36 to 4.99)	*p* = 0.009 *p* = 0.068 *p* = 0.004
**Number of dependents**	0.97 (0.81 to 1.18)	*p* = 0.789
**Type of accommodation** Rented house compared to family house Rented room compared to family house	1.84 (0.37 to 9.27) 0.18 (0.02 to 1.46)	*p* = 0.206 *p* = 0.458 *p* = 0.109
**Travel time from residence to factory** 15 min to 1 h compared to less than 15 min More than 1 h compared to less than 15 min	1.10 (0.69 to 1.76) 1.15 (0.60 to 2.20)	*p* = 0.892 *p* = 0.693 *p* = 0.676
**Working at hometown** Factory at hometown compared to not	0.74 (0.46 to 1.19)	*p* = 0.215
**Job title**		
Machine operator compared to Helper Quality controller compared to Helper Supervisor compared to Helper Other position compared to Helper	1.60 (0.91 to 2.81) 1.02 (0.43 to 2.39) 1.30 (0.49 to 3.45) 1.51 (0.53 to 4.34)	*p* = 0.467 *p* = 0.101 *p* = 0.968 *p* = 0.605 *p* = 0.442
**Monthly income category**		
Less than Rs. 25,000 compared to more than Rs. 25,000	0.87 (0.50 to 1.50)	*p* = 0.613
**Salary interval category**		
Daily or piece work earners compared to weekly or monthly earners	5.02 (1.54 to 16.33)	*p* = 0.007
**Level of work experience**		
1–10 years compared to less than 1 year More than 10 years compared to less than 1 year	1.23 (0.75 to 2.03) 1.05 (0.49 to 2.21)	*p* = 0.684 *p* = 0.417 *p* = 0.907
**Engaging in extra duty work** Yes compared to no	0.93 (0.59 to 1.48)	*p* = 0.768
**Full-time or part-time employed** Full-time compared to part-time	0.94 (0.55 to 1.62)	*p* = 0.835
**Permanent or casual worker** Permanent compared to casual	1.23 (0.65 to 2.30)	*p* = 0.528
**Time spent daily on watching television**		*p* = 0.336
Less than 1 h compared to none 1 to 3 h compared to none More than 3 h compared to none	0.81 (0.47 to 1.38) 0.78 (0.42 to 1.45) 0.15 (0.02 to 1.25)	*p* = 0.431 *p* = 0.428 *p* = 0.080
**Time spent daily on social media**		*p* = 0.307
Less than 1 h compared to none 1 to 3 h compared to none More than 3 h compared to none	1.10 (0.69 to 1.78) 1.49 (0.69 to 3.21) 3.44 (0.80 to 14.80)	*p* = 0.684 *p* = 0.305 *p* = 0.096
**Substance use** Presence compared to absence	1.07 (0.52 to 2.20)	*p* = 0.846
**Currently being pregnant or not** Yes compared to not relevant (male) No compared to not relevant (male)	0.98 (0.27 to 3.59) 1.22 (0.67 to 2.20)	*p* = 0.773 *p* = 0.979 *p* = 0.514
**Chronic physical illness** Presence compared to absence	2.91 (1.47 to 5.76)	*p* = 0.002
**History of depression** Presence compared to absence	7.85 (2.55 to 24.18)	*p* < 0.001
**History of attempted suicide** Presence compared to absence	7.29 (3.04 to 17.51)	*p* < 0.001
**Family history of mental illness** Presence compared to absence	2.91 (1.21 to 7.01)	*p* = 0.017

**Table 3:** Results of logistic regression analysis for psychological morbidity

Final Fitted Predictor Variables	Odds Ratio (95% Confidence Interval)	Statistical Significance
**Salary interval category** Daily or piece work earners compared to weekly or monthly earners	6.75 (2.05 to 22.24)	*p* = 0.002
**Chronic physical illness** Presence compared to absence	2.98 (1.45 to 6.11)	*p* = 0.003
**History of depression** Presence compared to absence	4.82 (1.44 to 16.15)	*p* = 0.011
**History of attempted suicide** Presence compared to absence	6.14 (2.45 to 15.39)	*p* < 0.001

After controlling for the other three variables in the model, garment factory workers that get paid daily or by the number of pieces they finish have 6.75 (95% C.I: 2.05 to 22.24) times higher odds of having a psychological morbidity compared to those earning monthly or weekly salaries. In comparison to individuals that did not report having attempted suicide in the past, those with such a history were 6.14 (95% C.I: 2.45 to 15.39) times more likely to be positive for psychological morbidity as assessed by GHQ-12. Similarly, having a history of depression accounted for about 5 times higher odds, and having a chronic illness lead to almost 3 times higher odds of showing psychological morbidity in garment factory workers (Table 4).

**Table 4:** Results of bivariate analysis for associated factors for depression

Potential Predictor Variables Considered	Unadjusted Odds Ratio (95% Confidence Interval)	Statistical Significance
**Age**	1.00 (0.97 to 1.03)	*p* = 0.980
**Gender** Female compared to male	2.03 (0.83 to 4.94)	*p* = 0.119
**Highest level of education** Above Ordinary Level compared to up to Ordinary Level	0.63 (0.33 to 1.19)	*p* = 0.150
**Marital status** Never married compared to currently married Separated, divorced or widowed compared to currently married	1.13 (0.61 to 2.10) 3.23 (1.57 to 6.65)	*p* = 0.005 *p* = 0.708 *p* = 0.002
**Number of dependents**	1.01 (0.79 to 1.28)	*p* = 0.950
**Type of accommodation** Rented house compared to family house Rented room compared to family house	0.97 (0.11 to 8.48) 0.49 (0.06 to 3.87)	*p* = 0.794 *p* = 0.981 *p* = 0.497
**Travel time from residence to factory** 15 min to 1 h compared to less than 15 min More than 1 h compared to less than 15 min	1.36 (0.74 to 2.52) 1.26 (0.54 to 2.93)	*p* = 0.614 *p* = 0.325 *p* = 0.590
**Working at hometown** Factory at hometown compared to not	1.16 (0.62 to 2.18)	*p* = 0.647
**Job title**		*p* = 0.402
Machine operator compared to Helper Quality controller compared to Helper Supervisor compared to Helper Other position compared to Helper	1.55 (0.75 to 3.17) 0.71 (0.21 to 2.38) 0.88 (0.23 to 3.47) 0.73 (0.15 to 3.59)	*p* = 0.234 *p* = 0.575 *p* = 0.859 *p* = 0.696
**Monthly income category** Less than Rs. 25,000 compared to more than Rs. 25,000	1.60 (0.72 to 3.54)	*p* = 0.245
**Salary interval category** Daily or piece work earners compared to weekly or monthly earners	2.05 (0.62 to 6.74)	*p* = 0.239
**Level of work experience**		*p* = 0.815
1–10 years compared to less than 1 year More than 10 years compared to less than 1 year	1.12 (0.59 to 2.14) 1.35 (0.54 to 3.35)	*p* = 0.724 *p* = 0.523
**Engaging in extra duty work** Yes compared to no	0.56 (0.32 to 0.99)	*p* = 0.044
**Full-time or part-time employed** Full-time compared to part-time	1.10 (0.54 to 2.24)	*p* = 0.788
**Permanent or casual worker** Permanent compared to casual	1.64 (0.67 to 4.02)	*p* = 0.279
**Time spent daily on watching television**		*p* = 0.774
Less than 1 h compared to none 1 to 3 h compared to none More than 3 h compared to none	0.82 (0.42 to 1.59) 0.73 (0.33 to 1.61) 0.41 (0.05 to 3.41)	*p* = 0.551 *p* = 0.433 *p* = 0.407
**Time spent daily on social media**		*p* = 0.781
Less than 1 h compared to none 1 to 3 h compared to none More than 3 h compared to none	0.79 (0.42 to 1.49) 1.14 (0.44 to 2.96) 1.58 (0.31 to 8.13)	*p* = 0.467 *p* = 0.788 *p* = 0.582
**Substance use** Presence compared to absence	0.99 (0.39 to 2.49)	*p* = 0.982
**Currently being pregnant or not**		*p* = 0.286
Yes compared to not relevant (male) No compared to not relevant (male)	1.67 (0.30 to 9.37) 2.05 (0.84 to 4.99)	*p* = 0.562 *p* = 0.116
**Chronic physical illness** Presence compared to absence	3.42 (1.66 to 7.05)	*p* = 0.001
**History of depression** Presence compared to absence	6.34 (2.46 to 16.32)	*p* < 0.001
**History of attempted suicide** Presence compared to absence	11.75 (5.25 to 26.29)	*p* < 0.001
**Family history of mental illness** Presence compared to absence	4.71 (1.94 to 11.44)	*p* = 0.001

### Prevalence of depression

All of the 132 individuals suspected of having psychological morbidity were screened for depression using the BDI-II. Out of the possible score range of 0 to 63, the GHQ-12 positive participants in the study had BDI-II scores from 0 to 46 with a mean score of 17.18 (SD = 10.37). Out of the 132 screened workers, 64 (48.48%) had a score equal to or above the BDI-II cut-off value of 16. Assuming that all the participants that were negative for psychological morbidity were also negative for depression, the estimated prevalence of depression in the study was 16.80% (95% CI: 13.04–20.55%).

### Predictors of depression

The identification of independent predictors of depression was done using the same process as that used with psychological morbidity. In bivariate analysis, six out of the before mentioned candidate predictor variables showed a significant association with presence of depression. These variables were: marital status, whether or not engaging in extra duty work, presence of a chronic physical illness, history of depression, history of attempted suicide and family history of mental illness (See Table 4). When performing logistic regression analysis using these six variables presence of a chronic physical illness, history of attempted suicide and family history of mental illness ended up in the final fitted model. According to the results, garment workers with a suicide attempt history had 10.79 (95% C.I: 4.68 to 24.89) times higher odds (compared to those who had not reported such a history) of currently having depression, after adjusting for the effects of chronic physical illness and family history of mental illness. Having a chronic medical condition leads to 3.51 (95% C.I: 1.61 to 7.67) higher odds of depression, while those with mental illness in their family resulted in three times higher odds of depression when controlling for the other two variables (See Table 5).

**Table 5:** Results of logistic regression analysis for depression

Predictor Variables	Odds Ratio (95% Confidence Interval)	Statistical Significance
**History of attempted suicide** Presence compared to absence	10.79 (4.68 to 24.89)	*p* < 0.001
**Chronic physical illness** Presence compared to absence	3.51 (1.61 to 7.67)	*p* = 0.002
**Family history of mental illness** Presence compared to absence	3.03 (1.11 to 8.26)	*p* = 0.030

## Discussion

The study flags a history of attempted suicide and chronic physical illness as clear predictors of psychological distress and depressive illness in garment factory employees studied. Not surprisingly, a family history of mental illness was predictive of depressive disorder and a history of depressive disorder was predictive of psychological distress [20]. Interestingly, demographic and employment factors did not show an association with psychological distress or depressive disorder apart from daily or piecework earners compared to weekly or monthly wage earners who showed more psychological distress.

In a study conducted in Colombo district in 2007 the point prevalence of depression was recorded as 1.6% (95% C.I: 1.3 − 1.9%) [21], which was much lower than the rate in this study. Another community study on randomly selected adults residing in parts of Gampaha district found a point prevalence of 11.3% [19]. A prevalence rate of depression at 41.1% (95% CI, 38.7–44.5) was reported by a study in a primary care setting in the Post-conflict Northern Province [22]. In a study on police officers in Kandy district, the prevalence of depression was computed as 10.6% [23].

In this sense the prevalence of depression in this group of garment factory employees appears higher than in the general population. To what extent the female preponderance in this population contributed to this is not known. Considering the fact that this is a relatively young population the figure is certainly more than the population prevalence. However, the depressive disorder was based on the screening tool BDI II and not established by a qualified professional which would have been ideal to arrive at a reliable point prevalence.

The prevalence of depression however in this study was relatively lower compared to other studies among factory employees from the Asian region where it ranged from 18 to 26% [8–10].

Around one third the employees screened showed psychological morbidity. This figure is less than the psychological stress seen in 60.4% of workers in garment factories reported from a Bangladesh study [24]. This finding is supported by a meta-analysis that has reported that the psychological stress among garment factory workers in Sri Lanka is less compared to other countries in the South Asian region [5].

There are several possible reasons for the relatively low prevalence of depressive symptoms and psychological distress in this group of garment factory employees in Sri Lanka compared to other South Asian countries. It is noteworthy that this study may not be representative of the garment factory employees in other parts of Sri Lanka. Our study population in Hambantota representing around 10% of all garment factories in Sri Lanka, was living in close proximity to their families which is not the case in many other studies in Sri Lanka in other factory areas such as the Free Trade Zone in Katunayaka, a suburban area. Hence the majority of employees in this study did not have to face the adversities associated with factories in semi-urban settings in populous districts where many of the garment factory workers are migrants living in boardings. A larger study in Sri Lanka encompassing different areas in Sri Lanka has clearly shown that depressive symptoms were linked to relocation from their homes [13].

This study did not identify most of the occupation-related variables as independent predictors of depression or psychological distress. Studies conducted in other Asian countries reported many occupation-related variables as independent predictors. A systematic review in Asian countries found that ready-made garment factory workers had psychological vulnerabilities due to low wages, excessive workload, job insecurity, abusive language and feeling unsafe in the workplace [5]. A positive factor that may be considered as a reason for this difference would be the relatively better working conditions in Sri Lankan garment factories compared to their counterparts in Asia. The same systematic review by Kabir et al. reported that Sri Lankan garment workers appeared to be healthier than garment factory workers in other developing countries and their reported quality of life was better [5]. According to study from Sri Lanka included in this review, very few employees reported being subjected to emotional abuse, and none reported sexual or physical abuse at work [25]. Furthermore, the majority of workers in our study were permanent employees and had better job security.

It is heartening however that government occupational health services in Sri Lanka work with factory owners and employees to ensure a favorable work environment. There is a dedicated medical officer of environmental and occupational health who oversees the physical and psychological concerns of garment factory employees in the Hambantota district, appointed by the Regional Director of Health Services. Workers’ health records are maintained at the medical unit in each garment factory and occupational and Health Officers, and Occupational and safety officers are trained as counsellors in these garment factories. The employers themselves are aware of the psycho-social stressors of their employees and provide psychological services such as access to a counsellor [26]. These measures may have mitigated occupation related stress factors from impacting mental health directly.

### Limitations

It has to be accepted that the sample size, which was calculated to determine the prevalence, was not perhaps adequate to capture the socio-economic factors that were studied resulting in a type 1 error. Furthermore, the study instruments were based on self-reports. As discussed earlier, the majority of the study participants were locally resident and travelling from their own homes as is not the case with most suburban textile factory employees, limiting the generalisability of this study.

## Conclusions

The study shows that the prevalence of depression among garment factory workers studied in southern Sri Lanka is higher than in the general population but lower than in other South Asian countries. The vulnerability factors are those common to depressive disorder in general. Employers need to be aware of the higher degree of mental health issues and continue to address them to improve employee wellbeing and productivity through targeted support that addresses both mental and physical occupational health.

## Data Availability

The data sets used and/or analysed during the current study are available from the corresponding author on reasonable request.
